# Supplementary figures and images for: Chemical mutagenesis of Listeria monocytogenes for increased tolerance to benzalkonium chloride shows independent genetic underpinnings and off-target antibiotic resistance
Source: PLoS One. 2024 Jul 19;19(7):e0305663. doi: 10.1371/journal.pone.0305663 (PMC11259264; doi:10.1371/journal.pone.0305663)

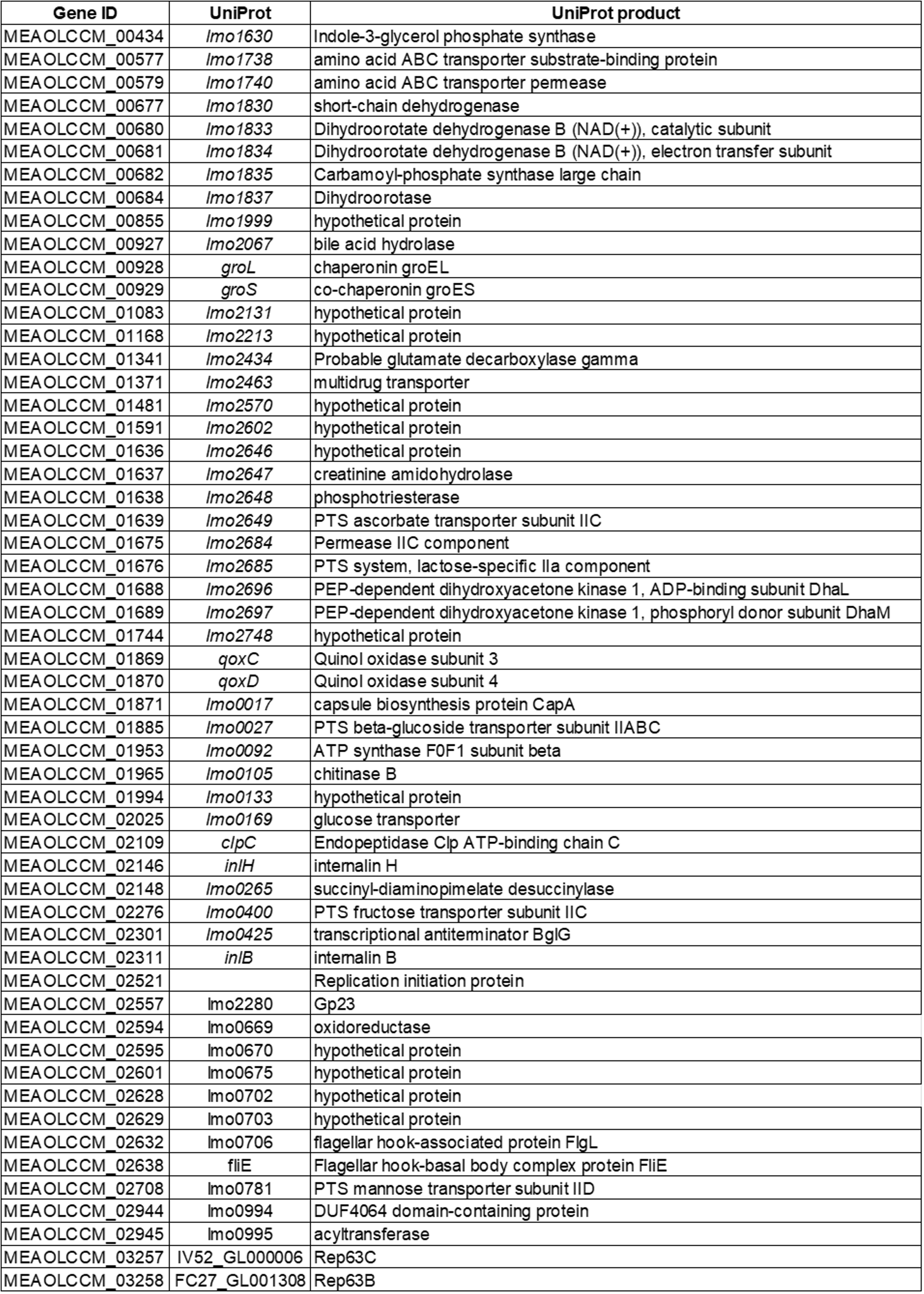

Supplement: S1 Fig — (TIF) [file pone.0305663.s001.tif]

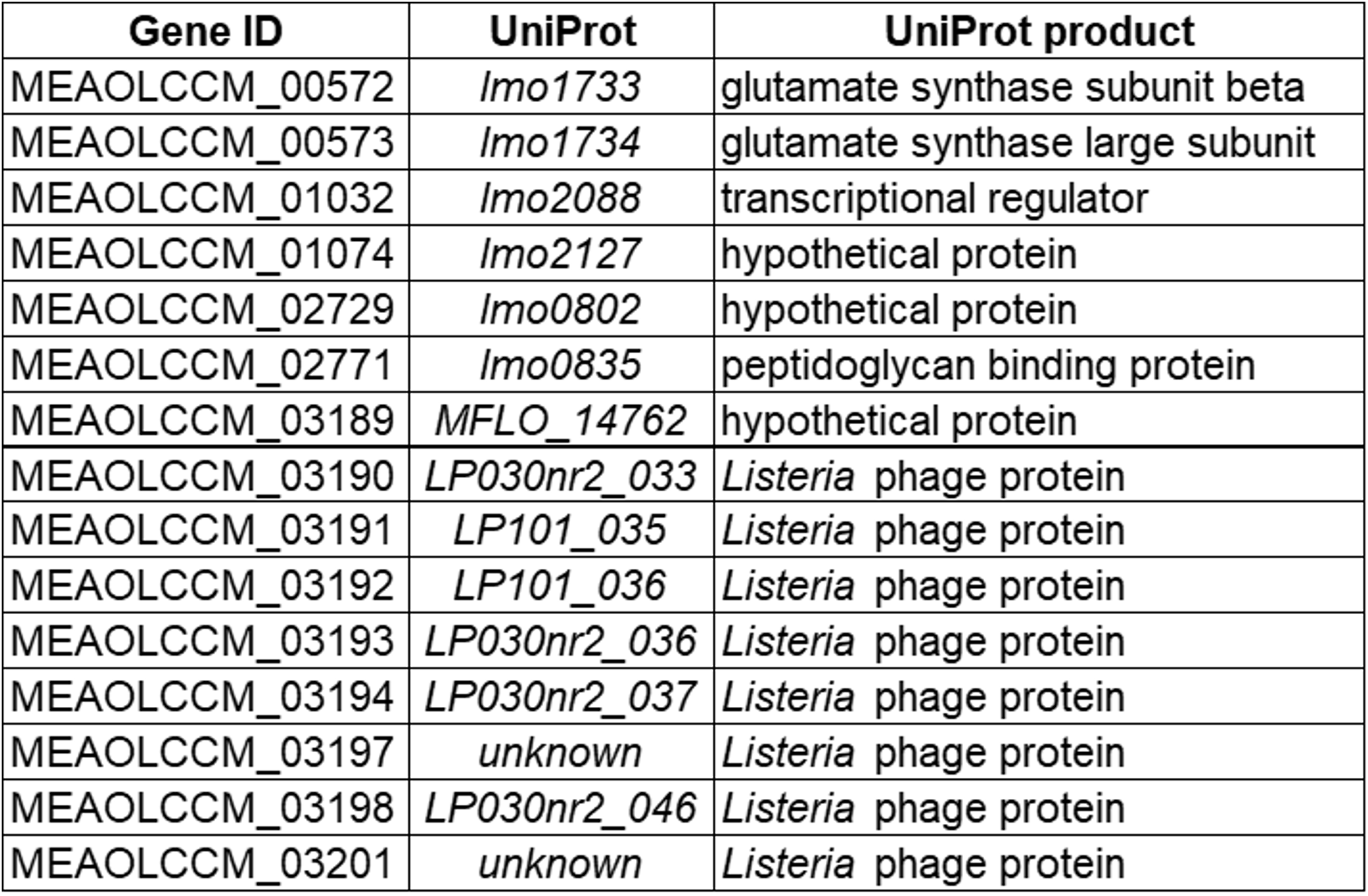

Supplement: S2 Fig — (TIF) [file pone.0305663.s002.tif]

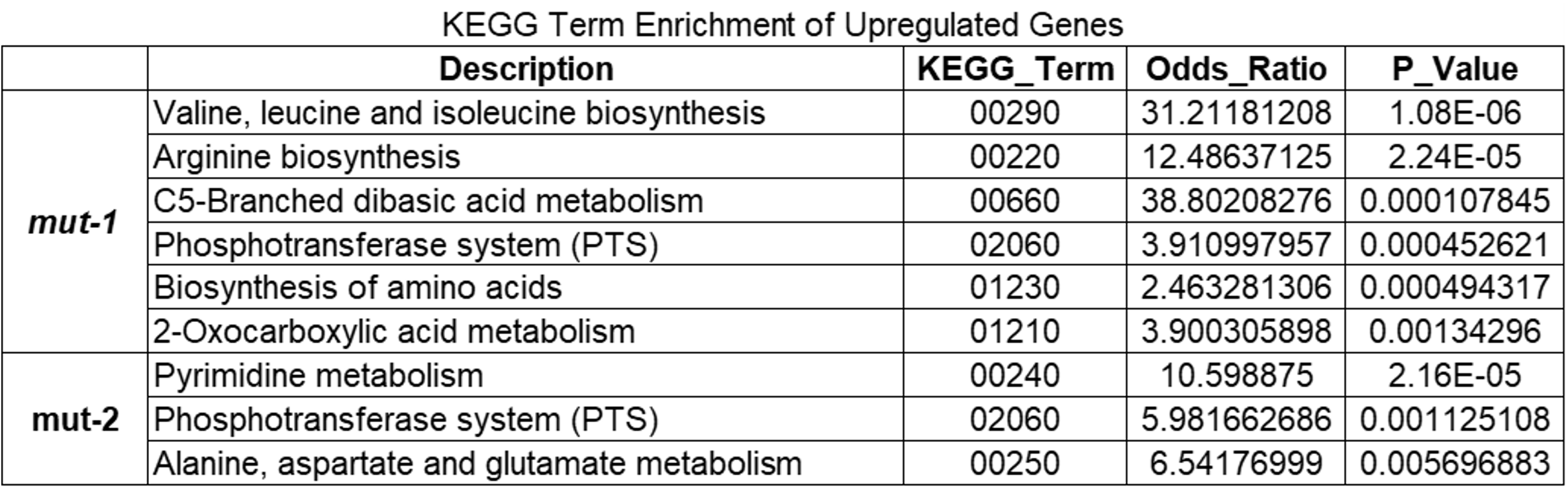

Supplement: S3 Fig — KEGG terms were assigned for the whole genome and enrichment analysis was performed using Fisher’s exact test to identify gene categories that were significantly up-regulated in the mutant strains (p-value < 0.01). (TIF) [file pone.0305663.s003.tif]

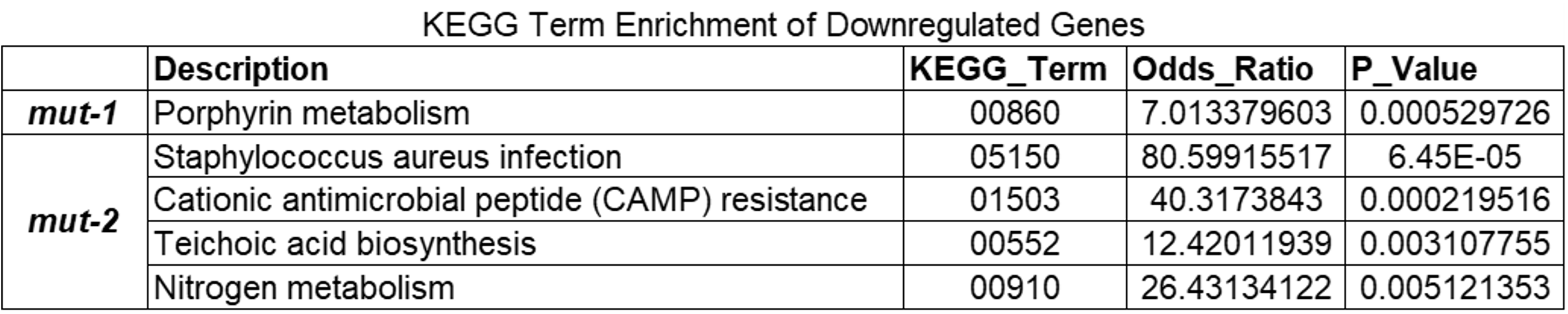

Supplement: S4 Fig — KEGG terms were assigned for the whole genome and enrichment analysis was performed using Fisher’s exact test to identify gene categories that were significantly down-regulated in the mutant strains (p-value < 0.01). (TIF) [file pone.0305663.s004.tif]
